# Unraveling the Molecular Pathways for Structure “Making” and “Breaking” by Ions in Water

**DOI:** 10.1021/jacs.5c10984

**Published:** 2025-10-04

**Authors:** Mischa Flór, Viktor Vorobev, Varun Mandalaparthy, Nico F. A. van der Vegt, Paul S. Cremer, Sylvie Roke

**Affiliations:** † Laboratory for Fundamental BioPhotonics (LBP), Institute of Bio-engineering (IBI), and Institute of Materials Science (IMX), School of Engineering (STI), and Lausanne Centre for Ultrafast Science (LACUS), 27218 École Polytechnique Fédérale de Lausanne (EPFL), Lausanne CH-1015, Switzerland; ‡ Department of Chemistry, 26536 Technical University of Darmstadt, Darmstadt 64287, Germany; § Department of Chemistry, 8082 Pennsylvania State University, University Park, Pennsylvania 16802, United States

## Abstract

Aqueous anions play a crucial role in chemical and biological processes. They are traditionally classified as “structure makers” or “structure breakers” based on their impact on the viscosity of electrolyte solutions. Until now, this behavior has been assumed to stem from a single restructuring mechanism of the hydrogen (H) bonding network of water, that could align with macroscopic properties. Correlated Vibrational Spectroscopy (CVS) measurements reveal that this is not the case. Rather, anions modify water–water H-bonds through multiple distinct pathways, with frequency shifts correlating with charge transfer, and intensity changes quantifying variations in the number of interacting/orientationally cross-correlated H-bonds. The different ways through which anions impact water structure can be explained in terms of Hard–Soft-Acid–Base theory. Hard anions only affect water H-bonds through electrostatics. By contrast, soft anions weaken the H-bonds via charge transfer but simultaneously increase their concentration. The two effects for soft anions nearly cancel each other out in terms of structure breaking/making, resulting in macroscopic behavior that is similar to hard anions in spite of dramatically different molecular-level effects.

## Graphical abstract

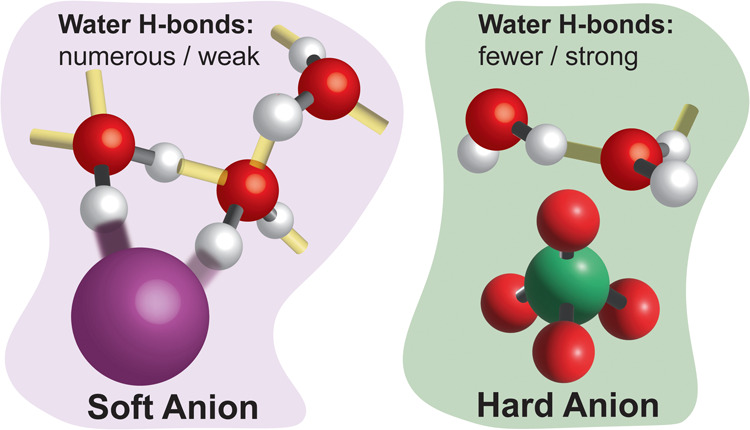

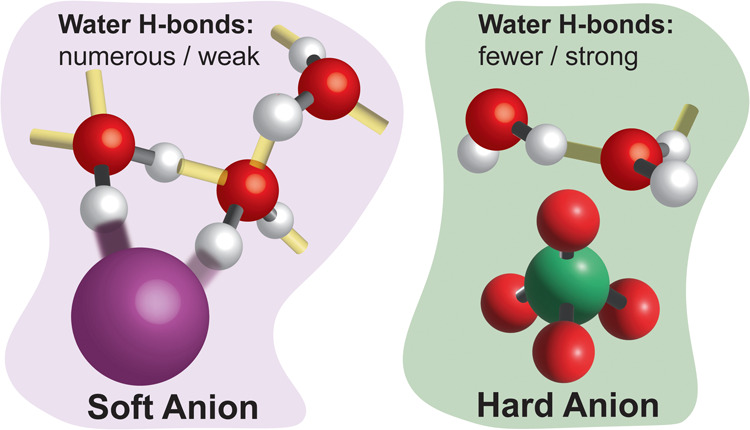

## Introduction

Ions are ubiquitous in nature and play a crucial role in chemical and biological processes through screening, binding and solvation. They influence enzyme activity,^1,2^ protein folding and unfolding.^3^ They are also essential for neural signal transmission^4^ and impact chemical equilibria and reactivity.^5^ In all these contexts, molecular interactions between anions and water molecules play a defining role. Anions are thought to change both the dynamics and local structure of the hydrogen (H)-bond network of water,^6–8^ and are commonly classified as either structure makers (“kosmotropic”) or structure breakers (“chaotropic”).^9^ Chaotropic anions are found to preferentially bind to hydrophobic portions of polymers and proteins, which enhances the polymer/proteins’ solubility in aqueous solutions.^1,10^ By contrast, kosmotropic anions induce hydrophobic collapse and protein folding through an excluded volume effect.^11^ This has led to the conclusion that chaotropes disrupt the H-bonding network, while kosmotropes enhance it.

The molecular-level interpretation of the behavior of chaotropic anions and their subsequent classifications as structure breakers and structure makers is typically inferred from macroscopic thermodynamic metrics, such as the viscosity B-coefficient,^12,13^ surface tension,^14^ the free energy of hydration,^15^ ion diffusion coefficients,^15^ lower critical solution temperatures for hydrophobic polymers,^16^ or the upper critical solution temperature for protein association.^11^ These experiments show homogeneous and often comparable trends when plotted for different anions. Because of these empirical trends, the ion-specific chaotropic behavior for anions has been attributed to a single specific molecular-level mechanism, primarily electrostatic in origin.^17–20^ Finding consistent evidence for this molecular level mechanism has been challenging as few techniques directly measure water–water H-bonds, even though numerous experimental and computational studies have been performed.^9^ O–H stretch vibrational spectroscopies,^21,22^ molecular dynamics (MD) simulations,^23^ and dielectric relaxation spectroscopy^24^ typically show minimal perturbations of water structure by anions. Meanwhile, neutron scattering,^25^ X-ray diffraction,^26^ and 2D IR spectroscopy^27^ suggest intermediate-range (nanometer-scale) ion-specific effects. Long-range effects of ions as probed via second-harmonic scattering at low concentrations are generally not ion-specific.^28^ The dynamics of water near anions has also been investigated using ultrafast IR spectroscopy, which reveals slowed H-bond dynamics and cation–anion cooperativity.^27,29,30^ Low-frequency vibrational spectroscopies such as THz and time-resolved THz-Raman spectroscopy have emerged as valuable tools for probing the hydration of ions. Low frequency spectra combined with MD simulations have revealed vibrational contributions from ions rattling in their hydration cage,^31,32^ signatures of short-lived anion-water bonding,^29,33,34^ and changes in the H-bonding network around ions.^7^ Additionally, solute–solvent polarization effects were shown to impact the THz spectral region.^35^ These spectrally broad modes are illustrated in Figure 1A and they all occur in the same frequency window. Disentangling these overlapping features is a great challenge. Thus, the question as to how ions impact the H-bond network of water on the molecular level is still open, as well as how structure breaking and making work at the molecular level.

**Figure 1:**
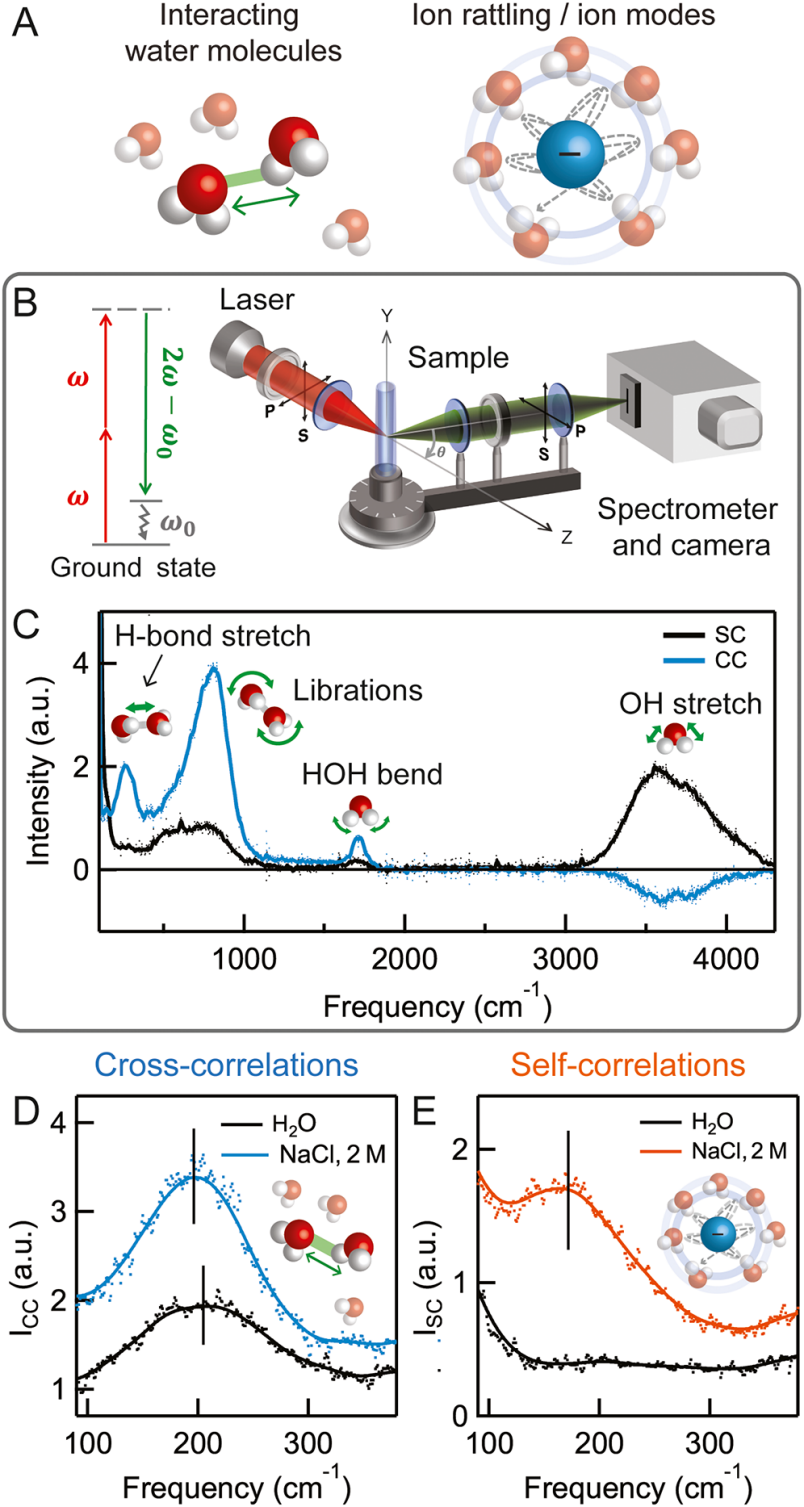
CVS applied to salt solutions. (A): Illustration of the contributions from electrolyte solutions. CVS separates interacting water contributions (left) from isotropic and direct ion contributions (right). (B): Illustration of the hyper-Raman scattering energy diagram and the experimental setup. P and S denote the polarization direction of light (horizontal and vertical, respectively). In our experiments, the scattering angle, θ, was set to 15°, which is close to the forward direction. (C): CVS spectra of pure water, showing the vibrational spectrum of orientationally cross-correlated water molecules (CC, blue) and that of individual water molecules (SC, black). The solid line represents a weighted running average. The vibrational modes of water are illustrated in the graph. (D): Measured CC spectra of water (black data) and a 2 M NaCl solution (blue data). The CC spectrum contains cross-correlated H-bond stretch modes, thus reflecting the H-bond network of water as modified by the solvated Na^+^ and Cl^–^ ions. (E): Measured SC spectra of water (black data) and a 2 M NaCl solution (red data). The SC spectrum contains contributions from modes with radial symmetry: ion rattling, intraionic, isotropic and single-molecule contributions. The Cl^–^ rattling mode is observed at 172 cm^–1^ and for Na^+^ it is expected to show up at 100 cm^–1^,^31^ which corresponds to the shoulder in this spectrum.

An answer to this question can be found by applying correlated vibrational spectroscopy (CVS)^36^ to the question of water structure. CVS is a novel hyper-Raman scattering-based method (HRaS, see Figure 1B) that disentangles the spectral contributions of interacting molecules from those of individual molecules. The method generally detects modes that are either IR or Raman allowed. CVS applies symmetry principles to HRaS, with spectra being recorded in specific polarization combinations and scattering angles (Figure 1B, and methods). Using this procedure, the correlation function of the induced nonlinear polarization can be separated into a self-correlated intensity *I*_SC_ and a cross-correlated intensity *I*_CC_. *I*_SC_ contains spectral information on only the isotropic, averaged response from individual molecules, while *I*_CC_ contains spectral information on orientational cross-correlations, that is, the collective response from interacting molecules. Figure 1C shows both types of water spectra, wherein the H-bond stretch mode, that is, the translational displacement of water molecules along the H-bond, which directly reports on the H-bond network of water, is isolated at ∼205 cm^–1^ in the *I*_CC_ spectrum. The anion-water and rattling cage modes appear in the *I*_SC_ spectrum. The *I*_CC_ spectrum contains sufficient information to determine both the number and strength of H-bonds between water molecules in electrolyte solutions. The frequency of the H-bond-stretch mode reports on the strength of H-bonds that are orientationally cross-correlated and thus represents interacting water molecules. The intensity reports on the concentration of such H-bonds, via a quadratic dependence.

Here, we employ CVS to measure how cross-correlated water–water H-bonds, and thus water–water H-bond interactions, respond to anions. The spectra show that the molecular mechanisms behind chaotropic behavior vary, showing two distinct molecular motifs, even among anions with similar macroscopic chaotropic effects. The observed behaviors involve either (1) significant charge transfer in combination with a relatively large increase in the number of correlated H-bonds or (2) very little charge transfer with much less pronounced changes in correlated H-bonds. This difference is explained in terms of the softness or hardness of the anion as described by hard and soft acid and base (HSAB) theory.^37,38^ Soft anions, such as I^–^, with a high polarizability display the first type of behavior. They significantly increase the number of interacting H-bonds, while reducing their strength through charge transfer-induced covalency. By contrast, hard anions, such as ClO_4_^–^, with tightly bound donor electrons, display the second behavior and therefore disrupt water H-bonds primarily via electrostatic interactions. This leads to a small decrease in the number of interacting H-bonds with a minimal influence on H-bond strength. Despite the contrasting mechanisms between soft and hard anions, compensatory effects in the first case with halides still lead to similar macroscopic chaotropic behavior for I^–^ and ClO_4_^–^. Results with F^–^ and SO_4_^2–^ suggest that the second mechanism can be extended to kosmotropic anions as well. These findings provide a molecular-level framework for understanding anion-specific effects on water structure.

## Results

### Ion-Specific Changes to the H-Bond Network of Water

#### Spectroscopic Studies

Figure 1D,E shows CVS spectra of a 2 M NaCl solution, where *I*_SC_ and *I*_CC_ are plotted separately. The methods section which includes details concerning CVS, is provided in the Supporting Information (SI), Section S1. Analytical derivations of CVS are provided in Section S2. Aqueous NaCl solutions exhibit rattling cage modes for Cl^–^, and Na^+^ ions, which correspond to isotropic motions of ions in water cavities. The *I*_SC_ spectrum for 2 M NaCl in Figure 1E shows the rattling cage mode at 172 cm^–1^, and a shoulder below 100 cm^–1^, consistent with THz spectroscopy results interpreted by computational analysis.^31^ More significantly, the *I*_CC_ spectrum (Figure 1D) in the same spectral range captures the orientational water–water correlations. Specifically, the cross-correlated peak at 193 cm^–1^ is red-shifted by 12 cm^–1^ and strongly increased in intensity compared to neat water (205 cm^–1^). This means that chloride changes both the strength and number of orientationally correlated water–water H-bonds.^36^ The ability to directly measure changes in H-bond strength and the number of correlated H-bonds, via the *I*_CC_ spectrum, provides a powerful tool to answer questions concerning how anions change the molecular level water structure. We have obtained *I*_CC_ spectra from solutions of NaX salts whereby X is, thiocyanate (SCN^–^), iodide (I^–^), bromide (Br^–^), chloride (Cl^–^), fluoride (F^–^), nitrate (NO_3_^–^), perchlorate (ClO_4_^–^), sulfate (SO_4_^2–^) and hydroxide (OH^–^). All solutions were made at a concentration of 2 M, except NaF, which was prepared at its solubility limit (∼0.9 M). The spectra are shown in Figure 2A. Figure 2B shows the corresponding frequency shifts for the H-bond stretch mode with respect to neat water (green bars) together with results from MD simulations (orange bars, discussed below), while Figure 2C shows the variation in the number of correlated H-bonds ΔN/N_H_^_2_^_O_, obtained from the square-rooted intensity normalized by the Bose-Einstein-correction factor (see SI for the details) .

**Figure 2:**
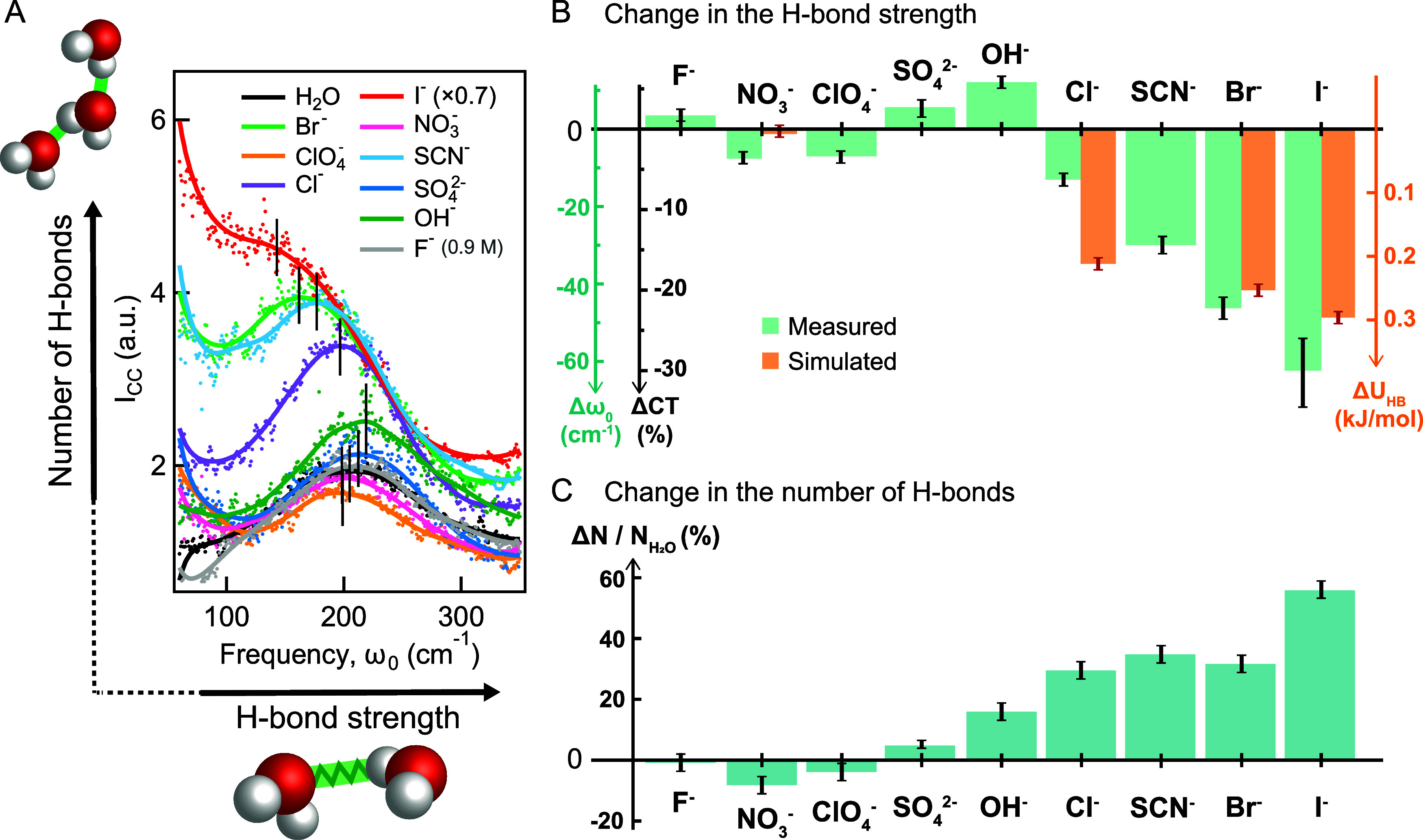
Ion-specific changes to the H-bond network of water measured with CVS. (A): Measured CC spectra of the H-bond stretching mode for different 2 M salt solutions. The solid lines represent weighted running averages. Strong anion-specificity is observed, with two distinct effects: The H-bond strength (*x*-axis, frequency) and the number of correlated H-bonds (*y*-axis, intensity) vary either significantly (I^–^, Br^–^, and SCN^–^) or very little (NO_3_^–^, ClO_4_^–^, SO_4_^2–^, F^–^). (B): Measured H-bond stretch frequency shift Δω_0_ and charge transfer shift (ΔCT) of the cross-correlated H-bond stretch modes upon the addition of salt (green) and simulated change in H-bond strength (orange) defined in the Methods section. ΔCT is estimated based on the proportionality between the change in charge density at the equilibrium position of the vibrational mode and the change in resonance frequency.^36^ (C): Measured change in number of orientationally cross-correlated (interacting) H-bonds, relative to pure water. Weaker H-bonds (in B) are more numerous (in C), and vice versa. The oxyanions NO_3_^–^ and ClO_4_^–^ show weak effects, as opposed to the other anions.

Figure 2 indicates two broad classes of spectral behavior: The *I*_CC_ spectra for I^–^, Br^–^, and SCN^–^ show dramatic increases in peak intensity, accompanied by pronounced red shifts. By contrast, the *I*_CC_ spectra of NO_3_^–^, ClO_4_^–^, F^–^, and SO_4_^2–^ show much smaller intensity and frequency changes. Among this latter class, the two chaotropic ions, NO_3_^–^ and ClO_4_^–^, show modest red shifts accompanied by slight decreases in peak intensity. By contrast, F^–^ and SO_4_^2–^ display slight blue shifts with SO_4_^2–^ possessing a very modest increase in intensity while F^–^ shows almost no change in intensity. Finally, there are two ions with somewhat intermediate behavior, Cl^–^ and OH^–^. The difference in the case of Cl^–^ is that it has a more modest red shift, but a pronounced increase in the number of correlated H-bonds. OH^–^ shows the greatest blue shift of any ion and yet leads to an increase in peak intensity.

The red- and blue shifts in orientationally correlated H-bonds have been found to correlate linearly with the amount of electronic charge transfer within the H-bond network.^36^ Moreover, an increase in the intensity scales with the square of the increase in the number of (interacting) H-bonds. The *I*_CC_ spectrum only probes orientationally cross-correlated H-bonds between water molecules. This response is produced by any pair of H-bonds^36^ that are orientationally correlated. In the SI, Section S3, analytical and experimental proofs are provided that the spectral contributions from water molecules with spherical symmetry around the ions are only present in the *I*_CC_ spectrum. Therefore, ion–water bonds and ion rattling modes are not expected to contribute to the *I*_CC_ spectrum, but only to the *I*_SC_ spectrum.

As can be seen, I^–^ ions shift the H-bond stretch mode center frequency by −62 cm^–1^ and increase the number of orientationally cross-correlated H-bonds by ∼56% compared to neat water. This number was obtained by correcting for a potential overlap with a shoulder of the second harmonic scattering peak, as shown in the SI, Section S4 and Figure S4. Iodide distributes ∼30% more electron density into the H-bond network of water compared to neat water, which might be expected to lead to an increase in the H-bond strength between water molecules. Consistently, small signatures of charge delocalization from iodide to water were identified through photoelectron spectroscopy and natural bond orbital analysis in concentrated NaI solutions.^39^ However, since there are 56% more orientationally correlated H-bonds between water molecules (Figure 2C), the increased charge is spread out among more interactions. As a result, each individual H-bond becomes weaker compared to those in pure water. For Cl^–^, an increase of ∼30% in interacting H-bonds together with a charge transfer increase of ∼6% is observed. Br^–^ and SCN^–^ behave in the same manner, but F^–^, NO_3_^–^, ClO_4_^–^, SO_4_^2–^ and OH^–^ do not.

F^–^, NO_3_^–^ and ClO_4_^–^ have much smaller frequency shifts (<5 cm^–1^), which translate into a minimal amount of charge transfer between the anions and water. Additionally, instead of increasing the number of interacting H-bonds, NO_3_^–^ and ClO_4_^–^ actually reduce this number, by ∼8% and ∼3% respectively. OH^–^ and SO_4_^2–^ also have a smaller influence on the H-bond stretch mode between water molecules. However, instead of having their strength decreased, they experience an increase in strength as manifested by blue shifts of ∼ +15 and ∼ +7.5 cm^–1^, respectively. Hydroxide increases the number of interacting H-bonds by ∼ +16% and sulfate by ∼ +5%.

Note that the increase in interacting H-bonds in any given electrolyte solution reports on the number of orientationally correlated H-bonds between water molecules. If the ions modify the structure of water in a way that only slightly perturbs the structure of bulk water, then the reported numbers are indicators of the number of H-bonds between water molecules. However, in the case of I^–^, where an increase of ∼56% is found, this number also potentially reflects a change in the H-bond network itself in the sense that it becomes more anisotropic, with the H-bonds deviating from radial symmetry. Note that the effect of Na^+^ is considered negligible compared to the anions,^40^ and cooperative anion–cation effects are also negligible in the recorded spectra, based on data from a concentration series (see SI Section S5 and Figure S5).

The results found in Figure 2 are quite curious. Fluoride, sulfate and hydroxide are traditionally classified as water “structure makers”, while the other six anions presented here are classified as “struture breakers”.^41^ It is certainly not anticipated that I^–^ should show the greatest increase in the number of H-bonds, while F^–^ shows almost no changes at all. In the discussion section we will come back to the meaning of chaotropic and kosmotropic behavior in light of the data that is presented.

#### Simulations

Solutions of NaNO_3_, NaI, NaBr, and NaCl were simulated using the Madrid 2019 ion^42^ force field combined with the TIP4P/2005 water model^43^ in GROMACS 2022.5.^44^ The systems consisted of 1111 water molecules and 40 ion pairs (2 M concentration). H-bonds were identified using a geometric criterion: a water–water H-bond was defined to form if the donor–acceptor oxygen distance (O_d_–O_a_) was <0.35 nm and the O_d_–H_d_···O_a_ angle was between 150° and 210°. The H-bond energy was derived from H_d_···O_a_ distance distributions.^45^ More details are provided in the Methods section. The results are shown in orange in Figure 2B (orange bars). We find that these four ions follow the same trend as found in the CVS measurements: decreases the average strength of H-bonds by 0.3 kJ/mol relative to pure water, followed by Br^–^, Cl^–^, and NO_3_^–^. Within the error bars, nitrate does not impact the strength of water H-bonds. While these calculations reproduce the results from the CVS measurements, they should be interpreted cautiously as contributions from charge transfer components and high frequency components of the polarization are neglected. The computational results in Figure 2B are consistent with the experimental results that show different classes of anions can have very different effects on the H-bonds network between water molecules. As with the experimental results, these behaviors do not align with general expectations if one groups the macroscopic observables from both types of ions. For example, NO_3_^–^ and I^–^ differ the most in Figure 2B, yet they are both classified as “structure breakers” with very similar macroscopic observables for their respective aqueous solutions.^12^ This suggests that there are distinct molecular mechanisms through which anions interact with the H-bonding network of water.

## Discussion

### HSAB Theory Explains the Distinct Molecular Behaviors

In order to link the molecular level observations (Figure 2) to macroscopic observables, we will consider concepts from HSAB theory.^37,38,46–48^ HSAB theory is used to predict the nature (covalent or electrostatic) and strength of bonding between a Lewis base (electron-pair donor) and a Lewis acid (electron-pair acceptor). In terms of anion-water interactions, the Lewis base is the anion, and the Lewis acid is water. HSAB theory qualitatively classifies compounds based on their electronic properties. Species with loosely held, highly polarizable electrons, such as Cl^–^, Br^–^ and I^–^, are classified as soft, while anions with tightly localized, less polarizable electrons, such as OH^–^, NO_3_^–^, ClO_4_^–^ and SO_4_^2^ are classified as hard.^49,50^ SCN^–^ is a hybrid anion, being soft on the sulfur side and hard on the nitrogen side.^51^ Like SCN^–^, water itself should be thought of as a hybrid molecule. Indeed, it is an amphoteric molecule, as it can both accept and donate electron pairs. The oxygen is rather hard, but the hydrogens are softer.

HSAB theory states that soft–soft interactions exhibit a strong covalent character due to electron sharing, whereas hard–hard interactions involve more weakly polarizable species and the corresponding interactions are predominantly electrostatic. Hard–hard and soft–soft interactions are typically favorable, whereas hard–soft interactions are less favorable. While HSAB theory is commonly expected to play a role in ion–water interactions,^47,52^ direct experimental tests and correlations with macroscopic quantities have mostly been lacking until now.^47^ The CVS results in Figure 2 can be correlated with macroscopic observables, like the viscosity B coefficient.^12^ As will be described below, an effective way to understand these behaviors is through HSAB theory.

The two distinct molecular behaviors that were identified from the data in Figure 2 can be best explained by invoking the softness or hardness of each anion. This works as follows: soft chaotropic anions, like I^–^, are expected to primarily interact with water through charge-transfer-induced covalent interactions.^51,53^ This idea is illustrated in Figure 3A where just one OH group from water points toward the anion, while the second one points toward another water molecule.^6^ The OH bond pointing toward the anion receives partial electronic charge from it. This soft interaction, in turn, leads to a sharp increase in the number of water–water interactions, but weakens them, as revealed by the orientationally cross-correlated H-bonds. By contrast, hard chaotropic anions, like NO_3_^–^ or ClO_4_^–^ interact with water primarily via electrostatics (i.e., ion-dipole interactions) with little charge transfer involved.^49^ This motif is illustrated in Figure 3B, whereby a water molecule can either orient its dipole toward the anion to create bifurcated H-bonding or point just one OH toward the anion.^54^ SO_4_^2–^ represent a good example of bifurcation^55,56^ while F^–^ is sufficiently small that just one OH points toward it.^57^ Because NO_3_^–^ and ClO_4_^–^ are considered chaotropic and have low charge density, the waters will be more weakly oriented with some not pointing very strongly toward the anion.^49^

**Figure 3:**
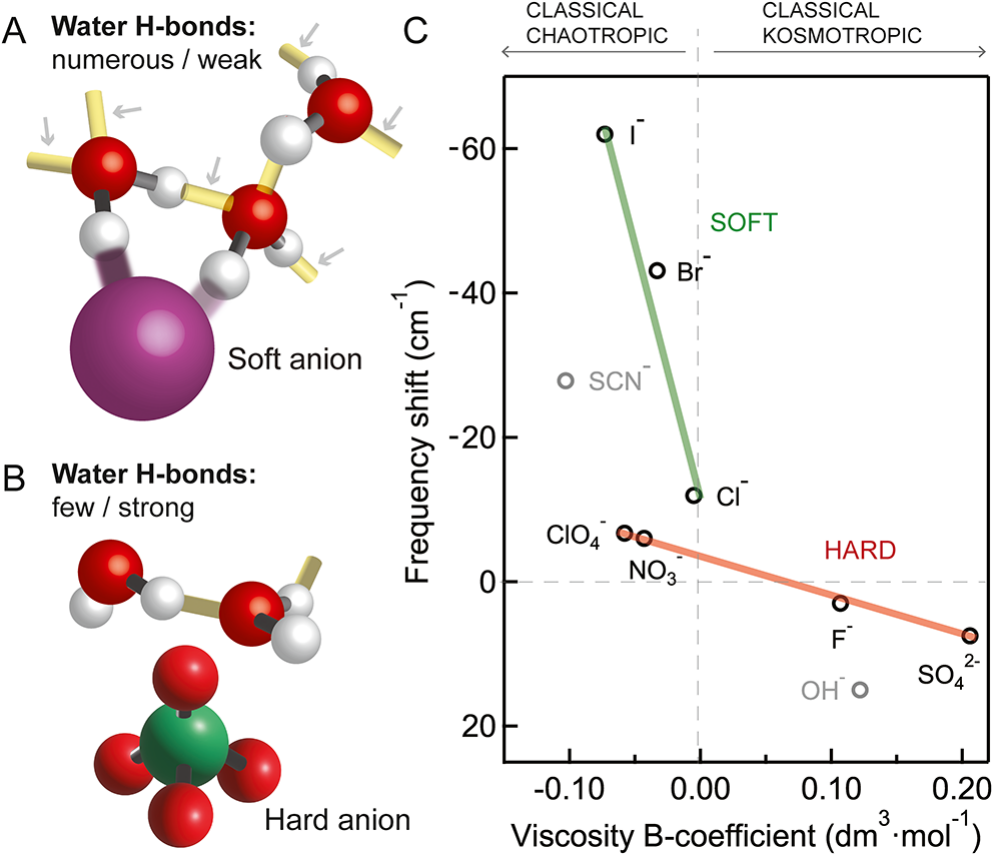
Soft and hard anions perturb water through different mechanisms. (A): Illustration of soft anion interactions. The arrows point to the H-bonds (in yellow) which are measured in the *I*_CC_ spectrum. In contrast, the purple bonds appear in the *I*_SC_ spectrum. Soft anions have easily distorted donor electrons, resulting in charge transfer-induced effects. This increases the number of water–water H-bonds, which are significantly weakened compared to pure water. (B): Illustration of hard anion interactions. Hard anions have tightly localized donor electrons which are difficult to share with surrounding water molecules. Instead, they disrupt hydration shell water through electrostatic effects, such as ion-dipole interactions, and only weakly influence the number and strength of interacting H-bonds between water molecules. (C): Measured frequency shift of the H-bond stretching mode between interaction water molecules, representing the change in the strength of H-bonds (data from Figure 2B), as a function of dynamic viscosity obtained from refs 12,58 The ranking of anions following the viscosity B-coefficient is only relevant once anions are grouped following their softness/hardness. SCN^–^ is highly asymmetric in its interactions (half soft, half hard), which multiplies the macroscopic chaotropic effect. OH^–^ is fully part of the H-bond network and transfers significant charge to the H-bond network.

The data in Figure 2 provides molecular level evidence for the two models of anion-water interactions described above. Indeed, the hard anions (SO_4_^2–^, F^–^, NO_3_^–^, ClO_4_^–^, and OH^–^) only weakly disrupt the strength of surrounding water–water H-bonds without significantly changing the number of interacting bonds. By contrast, I^–^, Br^–^, Cl^–^, and SCN^–^ can be treated as softer anions and show both a weakening of the water interactions (Figure 2B) as well as a substantial increase in their number (Figure 2C). Further evidence for this interpretation can be obtained by investigating the temperature dependence of the cross-correlated H-bond stretch signatures (Figure S6). For ion–water interactions that are primarily electrostatic, the correlated H-bond stretch mode should be strongly correlated with temperature. However, a smaller temperature dependence would be expected for systems that involve charge transfer, since the interactions are more covalent in nature. Indeed, Na_2_SO_4_ solutions show a strong temperature dependence, while the ones from NaCl and NaSCN are much more modest.

### Connecting Molecular Level Behavior to Classical Structure Making and Breaking

Trends in macroscopic observables have led to the emergence of the “structure breaker” and “structure maker” terminology. While multiple criteria exist, the viscosity B-coefficient^12,13,18^ is the most widely accepted metric for classifying ions as either “structure makers” or “structure breaker”.^9,59^ Dynamic viscosity provides a measure of the resistance of a fluid to deformation by an external stress. For an aqueous electrolyte solution, it is described by the empirical Jones-Dole equation,^9,12,15^ which has 2 coefficients, A and B. The A-coefficient is postulated to be related to ion–ion interactions, and the B-coefficient is thought to reflect ion–water interactions. Electrolyte solutions with anions that have B < 0 (e.g., SCN^–^ < I^–^ < ClO_4_^–^ < NO_3_^–^ < Br^–^ < Cl^–^) are known as “structure breaker” (chaotropes), while anions with B > 0 (e.g., F^–^ < OH^–^ < SO_4_^2–^) are classified as “structure makers” (kosmotropes).

The implicit assumption that underlies the terminology of structure breaking/making is that the resistance to motion in a liquid is caused by a single molecular level property. This is clearly not the case for the nine anions that were studied. Figure 3C shows the measured frequency shift for the H-bond stretch mode from Figure 2B as a function of the viscosity B-coefficient. Strikingly, I^–^, Br^–^, NO_3_^–^, SCN^–^ and ClO_4_^–^ have very similar viscosity B-coefficients, and yet, their molecular-level behaviors are very different. This difference can be understood in terms of HSAB theory.

As noted above, the two distinct molecular level behaviors attributed to the hardness and softness of the anions lead to separate trends in dynamic viscosity. First, the hard anions fall along the red line, revealing a quantitative relationship between frequency shift and viscosity B coefficient. Specifically, anions like SO_4_^2–^ that show a modest blue shift in the H-bond stretch frequency, lead to an increase in the solution viscosity, while anions like ClO_4_^–^ that show a very small red-shift lead to a more modest decrease in viscosity. For these hard anions, the shift in the H-bond stretch frequency can be related to the free energy of hydration as well as the charge density of the anion.^19,60^ This means that the extent of electrostriction imprinted onto water by the anion causes either a slight weakening or strengthening of the H-bonding network, which is directly reflected in the viscosity change of the solution. Significantly, this is also reflected in the change in the concentration of H-bonds, as reflected in Figure 2C. Figure S7 plots the charge density of the anions as a function of the measured frequency shift.

In contrast with the hard anions, the three more chaotropic halide anions do not fit on this trend line. Although one can tentatively draw a separate line through them, there are only three of them, so quantitative conclusions cannot be drawn with certainty. Nevertheless, several important qualitative conclusions can be made. First, very large changes in the red-shift of the water–water stretch frequency only lead to modest changes in the viscosity B-coefficient. This should be expected because the substantial weakening of the water–water interactions (Figure 2B) is nearly offset by the increase in the concentration of such interactions (Figure 2C). Next, there is a clear break between the behavior of I^–^, Br^–^, and Cl^–^ on the one hand, and F^–^ on the other. This distinction fits beautifully into the framework of HSAB theory, where the absolute hardness of an anion can be described by the coefficient, η, obtained empirically.^46^ Higher η-values are associated with a harder anion.^46^ The values for the four halide anions are 7.0, 4.7., 4.2, and 3.7, respectively, for F^–^, Cl^–^, Br^–^, and I^–^. The ability of an anion to transfer electron density depends on the Lewis acid that will accept the charge, which in this case is the H-bond from H_2_O. Although water itself has a hardness of η = 7.0, the H-bond is considered to be somewhat softer than the oxygen atom.^61^ What the data in Figure 3C unambiguously shows is that F^–^ is sufficiently hard to essentially exhibit no charge transfer. Note that F^–^ and Cl^–^ have typically been classified as hard anions, while Br^–^ is described as borderline, and only I^–^ is called soft.^47^ However, this labeling is somewhat misleading as it is based on absolute hardness values calculated from gas-phase properties for metal complexation. In aqueous electrolyte solutions, the extent of charge transfer specifically depends on the relative hardness of the anion compared to the OH bonds of water.^46^ Therefore, in aqueous solution, it appears that Cl^–^, Br^–^, and I^–^ are all sufficiently soft to result in chaotropic behavior, while only F^–^ is hard enough to cause kosmotropic behavior.

Significantly, there are two anions, SCN^–^ and OH^–^, that do not neatly fit on either the trend line for hard anions or the more tentative one associated with the three softer halides. Both of these behaviors are relatively easy to understand. Indeed, SCN^–^, has already been described by Pearson as being an example of a species that that has “local hardness”.^51^ More recently, Baer and Mundy have shown using DFT calculations that the nitrogen side is associated with stronger hydration (i.e., kosmotropic behavior) and the sulfur side with weaker hydration (i.e., chaotropic behavior).^62^ The softer, chaotropic side clearly dominates the behavior of SCN^–^, which gives rise to both a red shift in the H-bond stretch frequency and a negative viscosity B-coefficient value. In contrast with SCN^–^, OH^–^ is clearly a kosmotropic anion. Moreover, its H-bonding frequency blue-shift puts it below the trend line for hard anions. This is expected because, like SCN^–^, OH^–^ lacks a chemically uniform surface. There are three lone pairs on the oxygen atom that form a ring of charge that serves as a hydrogen bond acceptor for water, while the hydrogen atom side is a very weak H-bond donor.^63^ Based on these two exceptions, we would hypothesize that any anion that lacks chemical uniformity on its surface will not fit onto a simple trendline. Another interesting case is iodate (IO_3_^–^), as simulations indicate strong kosmotropic binding at iodine and weaker interactions at oxygen, which makes the anion behave as a cation.^64^ Due to its low solubility (∼0.45 M), NaIO_3_ was not directly comparable to other salts. Our CVS measurements at 0.4 M (Figure S8, SI) indicate a minimal redshift (∼1 cm^–1^) with no intensity change, indicating negligible charge transfer to water. This aligns with IO_3_^–^ behaving as a cation, resulting in no charge transfer.^64^ These examples show that, beyond the identified mechanisms, additional effects such as hydration symmetry, ion size, or specific chemical interactions may also contribute to the overall macroscopic behavior.

### Terminology of Structure Breaking (Chaotropy) and Structure Making (Kosmotropy) Revisited

As Figure 2 and 3 clearly show, the viscosity B-coefficient values for the various anions arise from a mixture of molecular-level physical properties. Therefore, the terms “structure breaking” (chaotropy) and “structure making” (kosmotropy) need to be reconsidered. The data in this work shows that charge transfer and classical electrostatic interactions are both relevant. Indeed, two effects associated with the H-bonding network can be considered structure making: the creation of additional correlated H-bonds (intensity increase in the water–water stretch), and an increase in their strength (blue shift). Structure breaking is unambiguously associated with the opposite: a decrease in the concentration of correlated H-bonds (intensity decrease), and a decrease in their strength (red shift).

I^–^ makes water structure because it increases the number of correlated H-bonds between water molecules but simultaneously breaks the structure because it weakens the H-bonds compared to neat water. In this case, the latter property is dominant, so I^–^ has classically been thought of as a water structure breaker. By contrast, NO_3_^–^ also reduces the strength of the H-bond network of the surrounding water (red shift, ‘breaker’), but it also decreases the number of correlated H-bonds (‘breaker’). As such, the two effects for the hard anions point in the same direction. Of course, ions can have more complex chemistry, which leads to more anomalous behavior for species like OH^–^ and SCN^–^. Based on the findings above, the anions can be categorized according to Table 1.

**Table 1:** Anions Considered According to Their Structure Making and Breaking Effects as Defined by How They Impact the H-Bond Network of Water

			effect on structure
anion	H-bond strength	# of correlated H-bonds	strength + #H-bonds
I^–^	↓	↑	Breaker + Maker
SCN^–^	↓	↑	Breaker + Maker
Br^–^	↓	↑	Breaker + Maker
Cl^–^	↓	↑	Breaker + Maker
ClO_4_^–^	↓	↓	Breaker + Breaker
NO_3_^–^	↓	↓	Breaker + Breaker
OH^–^	↑	↑	Maker + Maker
F^–^	↑	-	Maker + Maker
SO_4_^2–^	↑	↑	Maker + Maker

The table above shows that soft anions (I^–^, Br^–^, Cl^–^) have both “structure making” and “structure breaking” influences on the H-bond network, which ultimately leads to their limited influence on the viscosity of water. On the other hand, sufficiently hard anions are either double structure makers (F^–^, OH^–^, SO_4_^2–^) or double structure breakers (ClO_4_^–^, NO_3_^–^).

Under the framework of HSAB theory, water can be thought of as a ligand. For sufficiently soft anions, a structure reminiscent of a coordination complex is formed, in which a single OH bond from water orients toward the anion to maximize charge transfer (Figure 3A). By contrast, when anions are sufficiently hard, ligand binding does not occur (Figure 3B). Previously, the central atom of a coordination complex has usually been a cation, although the net charge on the complex could still be negative if the negatively charged ligands more than offset the positive charge on the central metal ion (e.g., [PtCl_6_]^2–^ or [Mo­(CN)_8_]^4–^). The difference in the Hofmeister chemistry of electrolyte solutions is that anionic coordination complexes with Cl^–^, Br^–^, and I^–^ use an anion as the central ion.

## Conclusions

Until now, it was assumed that anions with similar macroscopic effects have similar effects at the molecular level. It was thought that the chaotropic and kosmotropic effects arose from a single molecular level mechanism that could be directly correlated to macroscopic properties. Molecular level evidence in the H-bond network was available but incomplete and indirect, since low frequency H-bond stretch modes are difficult to isolate and access. By employing correlated vibrational spectroscopy (CVS) we directly monitored water–water H-bonds in electrolyte solutions using the isolated H-bond stretch mode of interacting water molecules. The strength and number of these water–water H-bonds in the presence of different anions, revealed that two molecular mechanisms underly the ways in which anions interact with water. Soft anions (Cl^–^, Br^–^, I^–^) affect H-bond strength through charge transfer, partially compensated by an increase in the number of orientationally correlated H-bonds, while hard anions (NO_3_^–^, ClO_4_^–^, SO_4_^2–^, F^–^) affect the H-bonding network through electrostriction. Two anions, SCN^–^ and OH^–^, show net soft and hard properties, respectively, but do not fit neatly onto simple trendlines with metrics like viscosity B-coefficients because they do not have sufficiently uniform chemical surfaces. This study advances our understanding of ion-specific effects in aqueous environments by revealing the underlying relationship between Hofmeister chemistry and HSAB theory. Future research could extend this framework to Hofmeister cations, which will enable the building of a molecular framework around H-bond dynamics in processes such as protein folding, enzymatic activity, and battery operation as well as material design.

## Supplementary Material



## Notes

Supporting text in Section S1 (methods), Section S2 (summary of CVS theory), Section S3 (ion–water modes appear in the self-correlated spectrum), Section S4 (influence of the second-harmonic scattering peak), Section S5 (nonadditivity in the *I*_CC_ excess spectrum), and Section S6 (temperature dependence); Supporting figures: Figure S1 (CVS of aqueous NaSCN solutions), Figure S2 (additivity in *I*_SC_ but not in *I*_CC_), Figure S3 (center frequency ω_0_ as a function of the anion mass), Figure S4 (excess *I*_CC_ spectrum for different salt solutions, as a function of concentration), Figure S5 (influence of the second-harmonic scattering peak), Figure S6 (temperature dependence of the H-bond stretching mode in different 2 M salt solutions), Figure S7 (charge density as a function of the frequency shift in the H-bond stretching mode), and Figure S8 (CVS of sodium iodate solution); Supplementary tables Table S1 (fitting parameters for NaBr and NaI solutions spectra) and Table S2 (extracted parameters from the *I*_CC_ spectra) (PDF)
