# Supplementary material for: Unraveling the Molecular Pathways for Structure “Making” and “Breaking” by Ions in Water
Source: J Am Chem Soc. 2025 Oct 4;147(41):37328–36. doi: 10.1021/jacs.5c10984 (PMC12532281; doi:10.1021/jacs.5c10984)
Supplement: Supplementary file 1 [file ja5c10984_si_001.pdf]

Supporting Information for

Unraveling the molecular pathways for structure ‘making’ and ‘breaking’ by ions in water

Mischa Flór[1], Viktor Vorobev[1], Varun Mandalaparthy[2], Nico F. A. van der Vegt[2], Paul S. Cremer[4,#], Sylvie Roke[1,#]

Corresponding authors: sylvie.roke@epfl.ch and psc11@psu.edu

Table of Contents:

S1. Methods

S2. Summary of CVS theory

S3. Ion-water modes appear in the self-correlated spectrum.

S4. Quantification and fittings

S5. Non-additivity in the I_{CC} excess spectrum

S6. Temperature dependence

S7. Molecular effects and their link to macroscopic observables

List of figures:

Figure S1. CVS of aqueous NaSCN solutions.

Figure S2: Additivity in $\boldsymbol{I_{SC}}$ but not in $\boldsymbol{I_{CC}}$.

Figure S3. The center frequency ω_0 as a function of the anion mass

Figure S4. Influence of the second-harmonic scattering peak.

Figure S5. Excess $\boldsymbol{I_{CC}}$ spectrum for different salt solutions, as a function of concentration.

Figure S6. Temperature dependence of the H-bond stretching mode in different 2 M salt solutions.

Figure S7 – Charge density as a function of the frequency shift in the H-bond stretching mode.

Figure S8 – CVS of sodium iodate solutions

List of tables:

Table S1 – Fitting parameters for NaBr and NaI solutions spectra.

Table S2 – Extracted parameters from the I_{CC} spectra.

S1. Methods

Correlated vibrational spectroscopy:

To generate Stokes-hyper Raman emission from a liquid, two photons with the same frequency ω interact with it. The electromagnetic field of the incident laser beam felt by a molecule v at position $\boldsymbol{r}_v$ is defined as: $\widetilde{\boldsymbol{E}}_v(\omega) \equiv \boldsymbol{E}(\omega)e^{i\boldsymbol{k}_1\cdot\boldsymbol{r}_v} + c.c.$, and $\boldsymbol{E}(\omega) = \boldsymbol{E}_0 e^{i\omega t}$. Upon elastic scattering, the photons are converted into a photon with wavevector $\boldsymbol{k}_0$, and a double frequency 2ω, which is scattered at an angle θ with respect to the incoming beam. Upon inelastic Stokes hyper Raman scattering the photons are converted into a frequency $2\omega - \omega_0$, whereby ω_0 is related to the first vibrationally excited state (having energy $\hbar\omega_0$, see Fig. 1B) in which the molecule is left after emission. The incoming field induces a molecular polarizability $\boldsymbol{p}_v^{(2)}$, which is responsible for the emission of light having an intensity (I):

$$I \sim \left|\sum_v \boldsymbol{p}_v^{(2)}\right|^2 = \left\{\underbrace{\sum_v \left|\boldsymbol{p}_v^{(2)}\right|^2}_{SC\ term} + \underbrace{\sum_{v\neq v'} \boldsymbol{p}_v^{(2)} \boldsymbol{p}_{v'}^{*(2)} e^{i\boldsymbol{q}\cdot(\boldsymbol{r}_v - \boldsymbol{r'}_{v'})}}_{CC\ term}\right\} = I_{SC} + I_{CC} \qquad \text{(S1)}$$

The first term in Eq. S1 represents self-correlations of individual molecules, and leads to incoherent contribution to the total intensity (I_{SC}). This is incoherent light scattering. This type of emission is present for every molecule that has an anisotropic structure. The second term in Eq. S1, i.e., the double summation over v and v', represents cross-correlations between different molecules and leads to a coherent contribution to the total intensity (I_{CC}). The correlation between two different molecules also has a phase factor, $e^{i\boldsymbol{q}\cdot(\boldsymbol{r}_v - \boldsymbol{r'}_{v'})}$, in which $\boldsymbol{q}\cdot(\boldsymbol{r}_v - \boldsymbol{r'}_{v'})$ is the phase difference between the emitted field of the two molecules v and v', and $\boldsymbol{q} \equiv \boldsymbol{k}_0 - 2\boldsymbol{k}_1$, the scattering wave vector. The second term brings insight into the specific liquid structure, as it reports on the orientational correlations of the molecules and therefore on the interactions between different molecules. The emission is thus composed of self-correlations (SC term, I_{SC}) and cross-correlations (CC term, I_{CC}) which can be separated by means of a symmetry analysis, summarized in S2. Applying this symmetry analysis to the spectrum of water, we previously showed that the I_{CC} spectrum contains only interacting modes (I_{CC}, blue trace Fig. 1C), of which the H-bond stretch mode located at 205 cm^{-1} is a prominent example. In Ref. [1] we also showed that the central frequency and shifts therein are proportional to changes in the charge transfer of partial electronic charge through H-bonds. The intensity of the H-bond stretch peak was shown to be proportional to the square of the number of cross-correlated H-bonds. By contrast, vibrational modes with radial or higher

symmetry appear in the I_{SC} spectrum. In the context of the present study, the ion-water and rattling cage modes are also relevant and these modes appear in the I_{SC} spectrum. To further support the assignment of rattling cage modes, and their appearance in the I_{SC} spectrum, and demonstrate the difference in spectral additivity between the I_{SC} and I_{CC} spectra, section S3 lists a series of experimental results containing a concentration series of ions that have intra-ionic vibrations (Fig. S1), an analysis of aqueous solutions containing HCl, NaOH, and NaCl (see Fig. S2), and analysis of the center frequency of the rattling cage mode peak in the I_{SC} spectrum of five different sodium salts in water (Fig. S3). The center frequency and the peak intensity of the H-bond stretching mode in the I_{CC} spectrum was obtained from the maximum intensity in the running average. The uncertainty was based on 11 consecutive measurements, yielding a standard deviation of 1.35 cm^{-1} in the frequency position and 1.2% in the intensity determination. This corresponds respectively to an uncertainty of 2.4 cm^{-1} and 2% with a confidence interval of 95%. For the cases of I^- and Br^-, the high intensity and low frequency requires to perform a multipeak fitting, resulting in higher uncertainties. The related details and fittings are shown in section S5.

CVS measurements

The laser setup and method is described in detail elsewhere[1]. Briefly, the CVS measurements used 5 ps laser pulses at 1028 nm with a 200 kHz repetition rate. Polarized (S or P) input pulses were focused down to a waist diameter of ~110 μm with 185 mW average power into a cylindrical glass cuvette (the sample cell) that contained the electrolyte solutions. The S or P analyzed HRaS spectra were recorded with 500 x 3 s acquisition time at θ=15° using an achromatic doublet with an acceptance angle of 7°, a spectrometer and an intensified charge coupled device (iCCD) camera. The reported central frequencies correspond to peak frequencies of the running average. The uncertainty on relative measurements was ±1 cm^{-1} with a 95% confidence interval. fs-Elastic second harmonic scattering was previously measured, which represents the averaged hyperpolarizability amplitude[2]. At 2 M, the difference between different ions does not exceed ~5 %. Therefore, the hyperpolarizability was assumed to be unchanged by the ions at the present concentration.

Sample preparation

H_2O was purified by a Milli-Q UF-Plus instrument from Millipore, Inc., and it has an electrical resistivity of 18.2 MΩ·cm. The salts (NaI, NaBr, NaSCN, NaCl, $NaClO_4$, $NaNO_3$) were used as received without further purifications, and were dissolved in ultrapure Milli-Q water to obtain the stock solutions. NaI 99.999% (metal basis) was purchased from abcr, NaBr , $NaNO_3$ 99.999% (trace metal basis) from Sigma Aldrich, NaCl 99.999% from abcr, NaSCN >

99.9900% (trace metals basis) from Sigma Aldrich, $NaClO_4$ 99.9% (trace metal basis) from Sigma Aldrich. The 2 M NaOH solution was freshly prepared before the measurements which did not take longer than 1 h. Within this timescale conductivity measurements have revealed a negligible change in the ionic strength of the solution.

Numerical simulations

We carried out simulations of various salt solutions ($NaNO_3$, NaI, NaBr, and NaCl) using the Madrid 2019 forcefield for the ions[3] in conjunction with the TIP4P/2005 water model[4] using GROMACS 2022.5[5]. We set up systems comprising 1111 water molecules and 40 ion pairs for a concentration of 2 M.

We began by energy minimizing the system via the steepest descent algorithm, followed by an equilibration in the NPT ensemble. This equilibration was run for 1 ns with a timestep of 2 fs. We accounted for electrostatics via the particle mesh Ewald method[6, 7], as implemented in GROMACS with a real space cutoff of 1 nm and a Fourier spacing of 0.1 nm. We calculated dispersion interactions via the Lennard-Jones potential with a cutoff of 1 nm and included tail corrections for the energy and pressure. We performed all simulations at 298 K and 1 bar pressure. We maintained the temperature via the Berendsen thermostat[8, 9] with a coupling constant of 2 ps and the pressure via the Parrinello-Rahman barostat[10] with a coupling constant of 2 ps. All angles were constrained via LINCS. [11]

Following the equilibration, we performed a 50 ns production run with the same settings as described for the equilibration except that we used the canonical velocity rescaling thermostat[12] with a coupling constant of 2 ps to maintain a temperature of 298K. For the analysis of the spectra (see below) all simulation parameters are identical to the production runs except that we perform 1000 independent simulation runs with a timestep of 1 fs. Each simulation is 800 ps long and we save configurations every 5 fs in an attempt to converge the desired correlation functions.

Hydrogen bonds were identified from our simulations using a standard geometric criterion. In particular, we define a water-water hydrogen bond to have formed between a pair of water molecules if the distance between the donor and the acceptor oxygens is less than 0.35 nm (O_d-O_a) and the angle between the O_d-H_d---O_a atoms is between 150 and 210 degrees. We calculated the energy of a hydrogen bond from the distribution of the H_d---O_a distances according to the following: [13]

$$U_{HB} = -25.36 \times 10^3 \times exp\ (-3.6 \times d_{H-O}).$$

The change in energy upon the addition of salt is written as

$$\Delta U_{HB} = U_{HB}(\text{salt solution}) - U_{HB}(\text{pure water}).$$

S2. Summary of CVS theory

In this section are summarize the theoretical derivations which led to the expressions that are central to CVS. The full detailed theoretical derivation can be found in the supporting material of Ref. [1].

The measured Stokes Hyper Raman intensity for a vibrational mode at frequency ω_0 is[1]

$$I(2\omega-\omega_0) \propto \left\langle \left| \boldsymbol{u}\cdot\boldsymbol{p}_{v,i}^{(2)}(2\omega-\omega_0)\right|^2 \right\rangle, \tag{S2}$$

where the unit vector $\boldsymbol{u}$ is in the direction of the detected polarization, onto which the frequency dependent induced second order dipole moment $\boldsymbol{p}_{v,i}^{(2)}$ is projected. The brackets $\langle\rangle$ represent an ensemble orientational average over all involved molecules and over the time duration of the laser pulses. Considering only the hyper-Raman Stokes contributions in the second order dipole moment, and that the incoming electric field is polarized in the direction of the unit vector $\boldsymbol{v}$,

$$\boldsymbol{u}\cdot\boldsymbol{p}_{v,i}^{(2)}(2\omega-\omega_0) = E_0^2 \textstyle\sum_{ijk} u_i v_j v_k \dot{\beta}_{v,ijk}^{(2)} \tilde{Q}(2\omega-\omega_0) \tag{S3}$$

Where $\dot{\beta}_{v,ijk}^{(2)} = \left(\frac{\partial\beta_{v,ijk}}{\partial Q}\right)_0$ are hyper-Raman tensor element derivatives with respect to the vibrational coordinate of interest (Q), $\beta_{v,ijk}$ being the hyperpolarizability elements, and where $\tilde{Q}(\omega_0)$ is defined as the Fourier transform of $Q(t)$, as $\tilde{Q}(\omega') = \int Q(t)e^{i\omega' t}\mathrm{d}t$.

The intensity then becomes:

$$\begin{aligned} I(2\omega-\omega_0) &\propto \left\langle E_0^4 \sum_{ijk}\sum_{i'j'k'} u_i v_j v_k u_{i'} v_{j'} v_{k'} \dot{\beta}_{v,ijk}^{(2)} \dot{\beta}_{v,i'j'k'}^{(2)} \left|\tilde{Q}(2\omega-\omega_0)\right|^2 \right\rangle \\ &= E_0^4 \left\langle \Delta\beta_v^2 \left|\tilde{Q}(2\omega-\omega_0)\right|^2 \right\rangle, \end{aligned} \tag{S4}$$

with E_0 the magnitude of the incoming electric field, defining:
$\Delta\beta_v^2 = \sum_{ijk}\sum_{i'j'k'} u_i v_j v_k u_{i'} v_{j'} v_{k'} \dot{\beta}_{v,ijk}^{(2)} \dot{\beta}_{v,i'j'k'}^{(2)}$ and $\Delta\beta_v = \sum_{ijk} u_i v_j v_k \dot{\beta}_{v,ijk}^{(2)}$.

The average in Eq. (S4) is[1]:

$$I(2\omega-\omega_0) = \langle\Delta\beta^2\rangle_{\mathrm{rot}} L(2\omega-\omega_0), \tag{S5}$$

Where "rot" indicates an orientational average and L accounts for the frequency-dependent line shape. Next, the HRaS intensity is calculated for the different polarization combinations PPP, SSS, SPP and PSS, using orientational averaging of the $\Delta\beta_v$ terms.

For SSS polarization, both the incoming and outgoing light are polarized perpendicular to the scattering plane, i.e., $\boldsymbol{u} = \boldsymbol{v} = \hat{\boldsymbol{Y}}$, a unit vector in the Y-direction. This results in a $\Delta\beta_v$ term that is independent of the scattering angle:

$$\Delta\beta_{\text{SSS}} = \sum_{ijk} Y_i Y_j Y_k \dot{\beta}^{(2)}_{v,ijk}. \quad \text{(S6)}$$

For SPP polarization, $\boldsymbol{v} = \hat{\boldsymbol{X}}$, a unit vector in the X-direction, and $\boldsymbol{u} = \hat{\boldsymbol{Y}}$, leading to:

$$\Delta\beta_{\text{SPP}} = \sum_{ijk} Y_i X_j X_k \dot{\beta}^{(2)}_{v,ijk} \quad \text{(S7)}$$

For PSS polarization, $\boldsymbol{v} = \hat{\boldsymbol{Y}}$ and $\boldsymbol{u} = \cos\theta\hat{\boldsymbol{X}} + \sin\theta\hat{\boldsymbol{Z}}$, which results in:

$$\Delta\beta_{\text{PSS}} = \cos\theta \left[\sum_{ijk} X_i Y_j Y_k \dot{\beta}^{(2)}_{v,ijk}\right] + \sin\theta \left[\sum_{ijk} Z_i Y_j Y_k \dot{\beta}^{(2)}_{v,ijk}\right] \quad \text{(S8)}$$

For PPP polarization both the incoming and outgoing light are polarized parallel to the scattering plane. If the incoming beam is polarized along the X-direction, $\boldsymbol{v} = \hat{\boldsymbol{X}}$. The outgoing polarization vector $\boldsymbol{u}$ depends on the scattering angle θ, which is defined such that θ is the angle between the incoming beam and the outgoing one. In this case, $\boldsymbol{u} = \cos\theta\hat{\boldsymbol{X}} + \sin\theta\hat{\boldsymbol{Z}}$. The resulting $\Delta\beta_v$ expression depends on the scattering angle:

$$\begin{aligned}\Delta\beta_{PPP} &= \sum_{ijk} \left[\cos\theta X_i X_j X_k + \sin\theta Z_i X_j X_k\right]\dot{\beta}^{(2)}_{v,ijk}, \\ &= \cos\theta \left[\sum_{ijk} X_i X_j X_k \dot{\beta}^{(2)}_{v,ijk}\right] + \sin\theta \left[\sum_{ijk} Z_i X_j X_k \dot{\beta}^{(2)}_{v,ijk}\right]\end{aligned} \quad \text{(S9)}$$

To compare the intensities in the polarization combinations SSS, SPP, PSS, and PPP there are four quantities that need to be rotationally averaged:

$$\langle\Delta\beta^2_{\text{SSS}}\rangle = \sum_{ijklmn} \langle Y_i Y_j Y_k Y_l Y_m Y_n\rangle \dot{\beta}^{(2)}_{v,ijk}\dot{\beta}^{(2)}_{v,lmn}, \quad \text{(S10)}$$

$$\langle\Delta\beta^2_{\text{SPP}}\rangle = \sum_{ijklmn} \langle Y_i X_j X_k Y_l X_m X_n\rangle \dot{\beta}^{(2)}_{v,ijk}\dot{\beta}^{(2)}_{v,lmn}, \quad \text{(S11)}$$

$$\langle\Delta\beta^2_{\text{PSS}}\rangle = \cos^2\theta \sum_{ijklmn} \langle X_i Y_j Y_k X_l Y_m Y_n\rangle \dot{\beta}^{(2)}_{v,ijk}\dot{\beta}^{(2)}_{v,lmn} + \sin^2\theta \sum_{ijklmn} \langle Z_i Y_j Y_k Z_l Y_m Y_n\rangle \dot{\beta}^{(2)}_{v,ijk}\dot{\beta}^{(2)}_{v,lmn} + \frac{1}{2}\sin(2\theta) \sum_{ijklmn} \langle X_i Y_j Y_k Z_l Y_m Y_n\rangle \dot{\beta}^{(2)}_{v,ijk}\dot{\beta}^{(2)}_{v,lmn}, \quad \text{(S12)}$$

$\langle \Delta\beta^2_{\mathrm{PPP}} \rangle = \cos^2\theta \sum_{ijklmn} \langle X_i X_j X_k X_l X_m X_n \rangle \dot{\beta}^{(2)}_{v,ijk} \dot{\beta}^{(2)}_{v,lmn} + \sin^2\theta \sum_{ijklmn} \langle Z_i X_j X_k Z_l X_m X_n \rangle \dot{\beta}^{(2)}_{v,ijk} \dot{\beta}^{(2)}_{v,lmn} +$
$\frac{1}{2}\sin(2\theta) \sum_{ijklmn} \langle X_i X_j X_k Z_l X_m X_n \rangle \dot{\beta}^{(2)}_{v,ijk} \dot{\beta}^{(2)}_{v,lmn},$ (S13)

These expressions are in their most general form and still account for both self-correlations, and cross-correlations.

For an isotropic medium, there are only self-correlations. In this case Eqs. S10-S13 can be simplified by averaging over rotations.
Averaging the molecules over all rotations in a fixed experiment, is mathematically equivalent to averaging over all possible ways in which the experimental apparatus can be oriented, with fixed molecules. For an isotropic medium, the four expressions in Eqs. S10-S13 can be simplified. First, for $\langle \Delta\beta^2_{\mathrm{PPP}} \rangle$, $\langle X_i X_j X_k Z_l X_m X_n \rangle = 0$ because Z appears as an odd power: if we were to send the incoming light beam in the opposite direction, along the $-Z$ axis, then this term would be negated. This also applies to $\langle \Delta\beta^2_{\mathrm{PSS}} \rangle$ where $\langle X_i Y_j Y_k Z_l Y_m Y_n \rangle = 0$, because X and Z appear as odd powers. Thus, we obtain:

$\langle \Delta\beta^2_{\mathrm{SSS}} \rangle = \sum_{ijklmn} \langle Y_i Y_j Y_k Y_l Y_m Y_n \rangle \dot{\beta}^{(2)}_{v,ijk} \dot{\beta}^{(2)}_{v,lmn},$ (S14)

$\langle \Delta\beta^2_{\mathrm{SPP}} \rangle = \sum_{ijklmn} \langle Y_i X_j X_k Y_l X_m X_n \rangle \dot{\beta}^{(2)}_{v,ijk} \dot{\beta}^{(2)}_{v,lmn},$ (S15)

$\langle \Delta\beta^2_{\mathrm{PPP}} \rangle = \cos^2\theta \sum_{ijklmn} \langle X_i X_j X_k X_l X_m X_n \rangle \dot{\beta}^{(2)}_{v,ijk} \dot{\beta}^{(2)}_{v,lmn} +$
$\sin^2\theta \sum_{ijklmn} \langle Z_i X_j X_k Z_l X_m X_n \rangle \dot{\beta}^{(2)}_{v,ijk} \dot{\beta}^{(2)}_{v,lmn},$ (S16)

$\langle \Delta\beta^2_{\mathrm{PSS}} \rangle = \cos^2\theta \sum_{ijklmn} \langle X_i Y_j Y_k X_l Y_m Y_n \rangle \dot{\beta}^{(2)}_{v,ijk} \dot{\beta}^{(2)}_{v,lmn} +$
$\sin^2\theta \sum_{ijklmn} \langle Z_i Y_j Y_k Z_l Y_m Y_n \rangle \dot{\beta}^{(2)}_{v,ijk} \dot{\beta}^{(2)}_{v,lmn}.$ (S17)

In addition, for an isotropic medium, rotating the coordinate system leaves the rotational averages identical. Considering a 90° rotation of the experiment around the Z axis, we obtain that $\langle Y_i Y_j Y_k Y_l Y_m Y_n \rangle = \langle X_i X_j X_k X_l X_m X_n \rangle$ and that $\langle X_i Y_j Y_k X_l Y_m Y_n \rangle = \langle Y_i X_j X_k Y_l X_m X_n \rangle$. For a 90° rotation around X, we obtain that $\langle Y_i X_j X_k Y_l X_m X_n \rangle = \langle Z_i X_j X_k Z_l X_m X_n \rangle$. Finally, rotating the coordinate system 90° around Y results in $\langle X_i Y_j Y_k X_l Y_m Y_n \rangle = \langle Z_i Y_j Y_k Z_l Y_m Y_n \rangle$. Introducing these equalities, we obtain the following non-zero quantities and properties:

$\langle \Delta\beta^2_{\mathrm{SSS}} \rangle$; $\langle \Delta\beta^2_{\mathrm{SPP}} \rangle = \langle \Delta\beta^2_{\mathrm{PSS}} \rangle$; and $\langle \Delta\beta^2_{\mathrm{PPP}} \rangle = \cos^2\theta \langle \Delta\beta^2_{\mathrm{SSS}} \rangle + \sin^2\theta \langle \Delta\beta^2_{\mathrm{SPP}} \rangle$ (S18)

where $\langle \Delta\beta_{\mathrm{SSS}}^2 \rangle$ and $\langle \Delta\beta_{\mathrm{SPP}}^2 \rangle = \langle \Delta\beta_{\mathrm{PSS}}^2 \rangle$ are independent of the scattering angle. For the intensities of the four polarization combinations PPP, PSS, PPS and SSS, we obtain:

$$I_{SPP}(2\omega - \omega_0) = I_{PSS}(2\omega - \omega_0) \quad \text{(S19)}$$

$$I_{PPP}(2\omega - \omega_0) = \cos^2(\theta) I_{SSS}(2\omega - \omega_0) + \sin^2(\theta) I_{SPP}(2\omega - \omega_0) \quad \text{(S20A)}$$

or

$$I_{PPP}(2\omega - \omega_0) - \cos^2(\theta) I_{SSS}(2\omega - \omega_0) + \sin^2(\theta) I_{SPP}(2\omega - \omega_0) = 0 \quad \text{(S20B)}$$

These expressions are thus valid for a sample with spatial isotropy. They can be used to measure both components, I_{SC} and I_{CC} of Eq. S1.

To do so, we first note that for a horizontal in-plane scattering geometry cross-correlations can be detected when the emitted SH or HRaS photons are (partially) polarized in the same direction as the scattering wavevector. When they are orthogonally polarized to the scattering wavevector only self-correlations can be probed [1, 14] That is, the polarization combinations PPP and PSS are detect both cross- and self-correlations in a nearly isotropic sample, while the SPP and SSS polarization combinations only detect self-correlation contributions. Thus, to disentangle the cross- and self-correlations to the intensity, both the polarization of the output beam and the scattering angle can be used.

For the self-correlations we can use:

$$I_{SC}(2\omega - \omega_0) = I_{SSS}(2\omega - \omega_0) + I_{SPP}(2\omega - \omega_0)\,, \quad \text{(S21)}$$

And for the cross-correlations we can use Eq. S20B, which leads to a vanishing response when there are only self-correlations, and a non-zero response when there are orientational cross-correlations for the vibrational mode of interest. Therefore, the cross-correlation intensity is:

$$I_{CC}(2\omega - \omega_0) = I_{PPP}(2\omega - \omega_0) - \cos^2(\theta) I_{SSS}(2\omega - \omega_0) + \sin^2(\theta) I_{SPP}(2\omega - \omega_0) \quad \text{(S22)}$$

These expressions have been validated experimentally in Ref. [1].

S3. Ion-water modes appear in the self-correlated spectrum.

The theory summarized in S2 allows us to make statements about the spectroscopic nature of vibrational modes in the sample. Vibrational modes that possess a radial symmetry, are present in the self-correlated spectrum, as the symmetry selection rules which lead to equations (S20) apply to them as well. For such modes $I_{PPP}(2\omega-\omega_0)-\cos^2(\theta)I_{SSS}(2\omega-\omega_0)+\sin^2(\theta)I_{SPP}(2\omega-\omega_0)=0$ and thus $I_{CC}(2\omega-\omega_0)=0$. The rattling cage modes of ions in water are described as damped harmonic oscillations that have a radial symmetry and are almost decoupled with their surrounding[15]. The first shell water-ion modes are also on average radial in symmetry, especially for spherical ions and contribute primarily to the I_{sc} spectrum[1]. Besides ion rattling and first-shell water contributions, the existence of 'mirrored water vibrations' is another contribution which emerged recently from numerical simulations[16]. These modes originate from solute-solvent polarization effects. In such modes, the ionic charge distribution fluctuates together with the charge distribution in the solvent, adding water-like vibrational features that originate from the ionic polarization, and not from the surrounding water. However, this coupling effect also results in intensity contributions to the self-correlated spectrum. Ref. [16] showed that close to the ion, the radially-integrated vibrational density of states is dominated by self-correlation contributions.

Next, we discuss experimental verifications of this expectation, by considering the following:

I. Vibrational modes of molecular ions in solutions
II. Additivity of spectral contributions to I_{SC} but not in I_{CC}
III. Mass dependence of the rattling cage mode frequency

Part of this analysis (items I and II) has appeared in a different form in Ref. [1].

<u>I. Vibrational modes of molecular ions in solutions</u>

Fig. S1 shows CVS spectra of aqueous 2 M NaSCN solutions, and water for comparison, with Fig. S1A showing the recorded PPP and SSS spectra of both liquids, Fig. S1B showing the I_{SC} and I_{CC} spectra for the electrolyte solution, and Fig. S1C showing the I_{SC} spectra obtained for a concentration series, scaled to the ion concentration. The Hyper Raman active vibrational modes that are visible in the spectra are: The H-bond stretch mode of water (205 cm-1, mostly in the I_{CC} spectrum), the broad libration band of water (~350-950 cm-1, mostly in the I_{CC} spectrum), and the intra-ionic stretch modes of the SCN- ion, which are the S=C=N bending mode (485 cm-1, in the I_{SC} spectrum), and the C=S stretch mode (~750 cm-1, in the I_{SC} spectrum)[17]. The decomposition in Fig. S1B confirms that in this spectral range, the intra-ionic modes of the ions themselves are uncorrelated. Fig. S1C shows that all concentrated weighted

I_{SC} spectra lie on top of each other, confirming that self-correlated spectral contributions are additive.

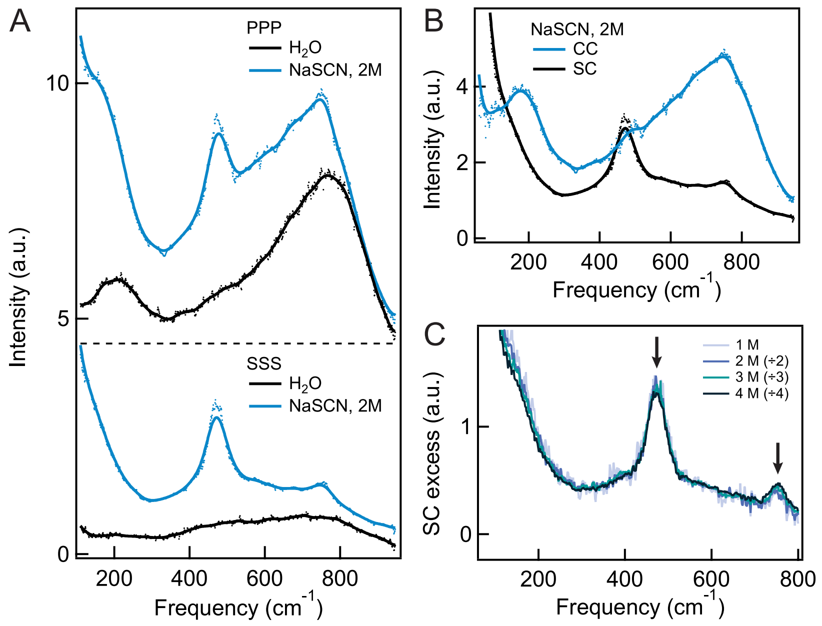

Figure S1: CVS of aqueous NaSCN solutions. A: Hyper Raman scattering spectra of water (back lines) and a 2 M NASCN solution (blue lines) recorded in PPP (top) and SSS (bottom) polarization combinations in the forward direction. **B**: I_{SC} and I_{CC} spectra of the 2 M electrolyte solution. **C**: The I_{SC} spectra obtained for a concentration series of NaSCN in water scaled to the ion concentration. The arrows indicate the intra-ionic stretch and bending modes of the SCN^- ion.

<u>II. Additivity of components in I_{SC} but not in I_{CC}</u>

Here we additionally confirm that the SC spectrum measures direct ion and solvation-shell contributions, in opposition to the CC spectrum which measures the distant water-water contributions.

Specifically, we know that:

(i) The individual ion contributions, as well as immediate first solvation shell water, are known to be additive[18].

(ii) The effect of ions to the H-bond network of water is not expected to be additive, due to the collective rearrangements of water molecules.

Therefore, if the separation is true, we should expect that SC spectra are additive while CC spectra are not. To experimentally verify the additivity, we have measured CVS spectra of HCl, NaOH, and NaCl solutions at a concentration of 2 M. Based on the properties (i) and (ii) above, we expect that the measured intensities will obey

$$I_{SC}((\mathrm{HCl})_{aq}) + I_{SC}((\mathrm{NaOH})_{aq}) - I_{SC}((\mathrm{NaCl})_{aq}) = I_{SC}(\mathrm{H_2O}) \qquad \text{(S23)}$$

In this equation, the ions that produce rattling modes and spherical hydration shells (ie., Cl^- and Na^+) will cancel out. In contrast, because of the non-additive effect of ions to the surrounding water, we expect that

$$I_{CC}((HCl)_{aq}) + I_{CC}((NaOH)_{aq}) - I_{CC}((NaCl)_{aq}) \neq I_{CC}(H_2O) \qquad \text{(S24)}$$

Fig. S2 shows both spectra, the I_{SC} in Fig. S2A and the I_{CC} in Fig. S2B. It can be seen that in Fig. S2A the orange (combination, left side of Eq. S22) and grey (water, right side of Eq. S22) spectrum are very close in shape/intensity, while the ones in Fig. S2B that correspond to the left (orange) and right side (grey) of Eq. S23 are not. This therefore supports the expectations of additivity.

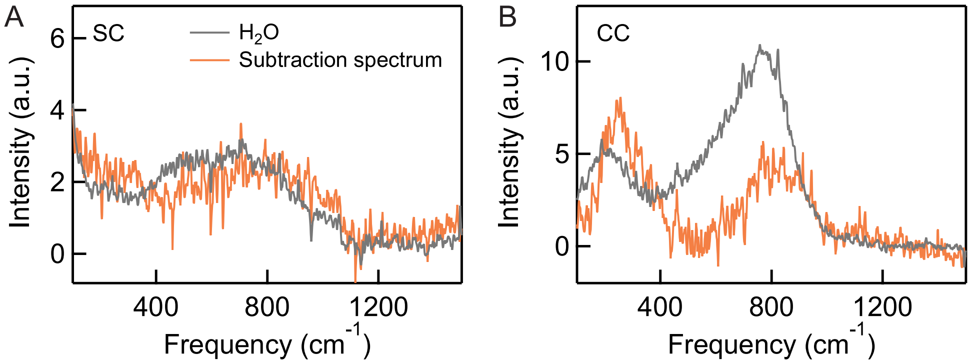

Figure S2: **Additivity in I_{SC} but not in I_{CC}. A**: I_{SC} spectrum of water (grey) and the combination spectrum of HCl + NaOH – NaCl (orange). By this addition, the spectral features of the ions are removed and the orange spectrum is very similar to the grey spectrum of pure water. **B**: I_{CC} spectra of water (grey) and the combination spectrum of HCl + NaOH – NaCl (orange). In this case the combination spectrum is different from that of water, as the spectral contributions stemming from the interactions are not additive.

III. Mass dependence of the rattling cage mode frequency

Next, we zoom in on the ion rattling cage modes. These modes refer to the vibrational motion of an ion within the "cage" formed by surrounding water molecules in its hydration shell. The ion undergoes oscillatory motions which are well described by a model of damped harmonic oscillations of the anions and cations within the water network[15], its frequency can therefore be written as $\omega_0 = \sqrt{k/\mu}$, where k is the force constant of the cage, and μ is the reduced mass. The latter is related to the ion mass. Thus, it is expected that the frequency of rattling cage modes is lower for heavier ions. Fig. S3A shows the measured center frequency (ω_0) of the main peak in the I_{SC} spectrum that corresponds to the ion rattling-cage mode for a series of anions, dissolved with Na^+ counterions at 2 M. Fig. S3B shows the center frequency of the main peak in the I_{CC} spectrum, which corresponds to the cross-correlated H-bond stretch modes for the same salts. The measured center frequency of the I_{SC} peak (Fig. S3A) is

correlated to the mass of the anion, as expected. This is not the case for the I_{CC} contribution, as shown in Fig. S3B. This observation confirms that ion rattling-cage modes contribute to the I_{SC} spectrum, but not to the I_{CC} spectrum.

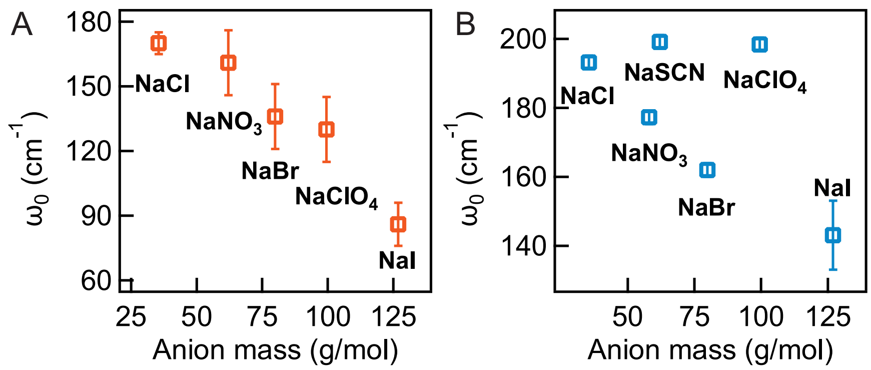

Figure S3 – The center frequency ω0 as a function of the anion mass. **A**: In the I_{SC} spectrum, the center frequency is correlated with the mass as expected for ion rattling-cage modes, which is, heavier ions rattle slower, at lower frequency. **B**: In contrast, in the I_{CC} spectrum there are no correlations with the molar mass of the anion. This is because the frequency of the peak is related to the strength of water-water H-bonds, which is not directly related to ionic mass.

S4. Quantification and fittings

The I_{CC} spectra of Fig. 2 show a clearly visible trend, but the cases of NaI and NaBr could be influenced by the decaying shoulder of the neighboring second-harmonic (SH) peak. However, other salt solutions such as NaCl are negligibly influenced by the SH shoulder, as shown in Fig.S5A by comparing the CC and SC spectra. For the cases of NaI and NaBr, to make sure the influence of the SH peak does not impact the proposed ranking of anions, we performed multipeak fittings using Voigt functions. Two peaks are expected in this region, the SH peak and the H-bond stretching mode. The SH peak is fixed at 0 cm^{-1} (by definition) and apparent center frequencies were used as initial guesses. The fittings converged to minimize the residuals. All fittings were performed using Gaussian fits in Igor Pro 9. Figs. S5B,C show the obtained fitted spectrum, together with the fitted SH peak and the fitted H-bond stretching mode. The obtained center frequency for the H-bond stretch mode is 162 cm^{-1} for the NaBr solution and 143 cm^{-1} for the NaI solution. The peak intensity is 388 a.u. and 599 a.u., respectively.

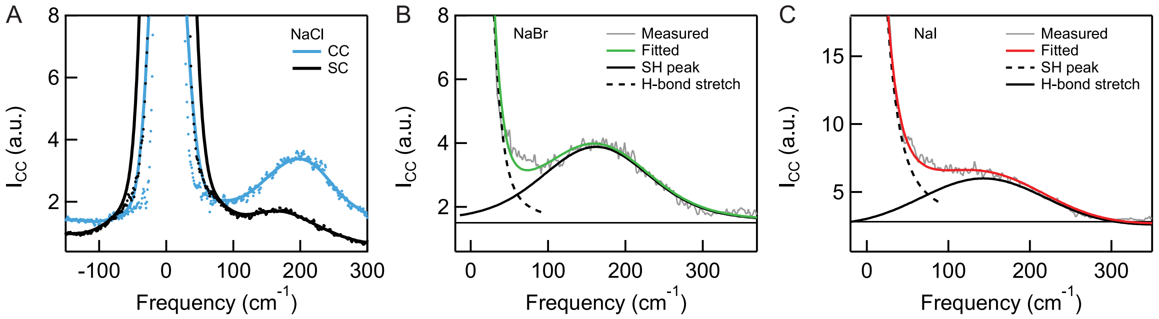

Figure S4 – Influence of the second-harmonic scattering peak. Spectra obtained for NaCl solutions (**A**), NaBr solutions (**B**) and NaSCN (**C**) solutions. The excess spectrum is divided by the salt concentration to identify non-additive contributions.

Table S1 provides the fitting parameters for those spectra. Note that uncertainties are around 2 cm^{-1} on the frequency values and 2.5% on the intensity values.

Table S1 – Fitting parameters for NaBr and NaI solutions spectra.

	Peak 1 frequency	Peak 1 width	Peak 1 intensity	Peak 2 frequency	Peak 2 width	Peak 2 intensity
NaBr	-1.8 cm^{-1}	26 cm^{-1}	2632 a.u.	162 cm^{-1}	96 cm^{-1}	403 a.u.
NaI	0.5 cm^{-1}	25 cm^{-1}	3950 a.u.	143 cm^{-1}	106 cm^{-1}	614 a.u.

Table S2 provides the extracted frequency and intensity parameters obtained from the spectra, together with the extracted numbers for charge transfer and number of orientationally-correlated H-bonds.

Table S2 – Extracted parameters from the I_{CC} spectra. Center frequency and calculated variation in charge transfer ΔCT estimated based on the proportionality between the change in charge density at the equilibrium position of the vibrational mode and the change in resonance frequency[1]. Measured intensity I_{CC} (a.u.), corrected intensity based on Eq. (S25), as well as the calculation of ΔN. The case of H_2O is shown as a reference.

Salt	ω_0 (cm^{-1})	ΔCT (%)	I_{CC} (a.u.)	Corrected I_{CC} (a.u.)	ΔN (%)
NaI	143	-0,30	614	304	56,0
NaBr	162	-0,21	403	217	31,8
NaSCN	177	-0,14	400	228	35,2
NaCl	193	-0,06	349	210	29,7
$NaNO_3$	198	-0,03	190	116	-3,4
$NaClO_4$	199	-0,03	174	106	-7,6
Na_2SO_4	213	0,04	217	138	5,3
NaOH	220	0,07	259	168	16,1
NaF	208	0,01	198	125	0,0
Pure H_2O (ref.)	205		200		

To obtain the number of orientationally-correlated H-bonds from the measured cross-correlation response I_{CC}, the measured intensity is first corrected using the prefactors in Eq. (S25) to account for the temperature-dependent vibrational occupation described by the Bose–Einstein distribution:

$$I(2\omega-\omega_0)=N^2\frac{32\pi^3}{3\hbar c^3}\frac{(2\omega-\omega_0)^4}{1-\exp\left[-\frac{\hbar\omega_0}{kT}\right]}\langle\Delta\beta_v^2\rangle \qquad \text{(S25)}$$

where N is the number of vibrational scatterers, ω is the laser frequency, ω_0 is the frequency if the vibrational resonance, $\langle\Delta\beta_v^2\rangle$ is the orientational average of the hyper-Raman tensor elements involved in the process, $\hbar$ is the reduced Planck constant, T is the temperature, k is the Boltzmann constant, c is the velocity of light. This operation was performed between the measured I_{CC} column and the "Corrected I_{CC}"column. ΔN is the variation (%) of the former column.

S5. Non-additivity in the I_{CC} excess spectrum

To ensure that our results are not strongly influenced by cooperative anion-cation effects at 2 M (e.g. ion-pairing, clustering effects), we have performed concentration series for different salt solutions. Cooperative ion effects can be probed via non-additive contributions to the low-frequency spectrum[19], in the excess spectrum (water spectrum subtracted to the solutions spectrum). Fig. S4 shows the measured excess cross-correlation spectrum, divided by the salt concentration. Compared to the maximum intensity, non-additive effects are relatively small, for the three salts shown here (NaCl, NaBr, NaSCN). We conclude that cooperative cation-anion effects do not impact our analysis on anion-water interactions measured at 2 M.

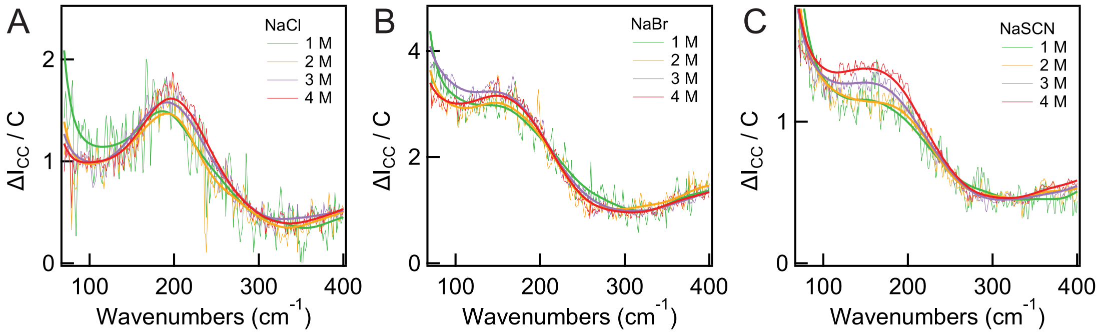

Figure S5 – Excess I_{CC} spectrum for different salt solutions, as a function of concentration. Spectra obtained for NaCl solutions (A), NaBr solutions (B) and NaSCN (C) solutions. The excess spectrum is divided by the salt concentration to identify non-additive contributions.

S6. Temperature dependence

In addition to the results presented in Fig.2, the effect of temperature on the H-bond stretching mode is investigated to provide further insight about the nature of ion-water interactions. Fig. S6 shows the measured I_{CC} spectrum in the H-bond stretch mode region, for three different salt solutions at 2 M: Na_2SO_4 (Fig. S6A), NaCl (Fig. S6B), and NaSCN (Fig. S6C), at three different temperatures, 273 K (blue line) 298 K (green line), and 353 K (red line). In this series of anions, SO_4^{2-} is a particularly hard anion, while SCN^- is soft, and Cl^- is in between. For interactions that are purely electrostatic, angle-averaged charge-dipole interactions are expected to decay with temperature following a 1/T dependence[20]. Therefore the I_{CC} response from water-water H-bonds surrounding hard anions, such as SO_4^{2-} should exhibit an intensity that decreases with temperature. Conversely, soft anions which result in charge transfer induced covalency, should not depend on the temperature.

In Fig.S6, we observe that upon heating, the three salt solutions display different effects. As expected, most important effects are observed for the SO_4^{2-} anions, where the intensity is reduced by 21% between 273 K and 353 K. For the Cl^- anions, the intensity reduction is only 11%, and for SCN^- anions, the intensity change is even negligible (~2% increase). In other words, the temperature dependence of the H-bond stretching mode seems to be also correlated with the hardness/softness of the anion, and confirms that the ion-water interactions primarily stem from the hardness/softness of the anion.

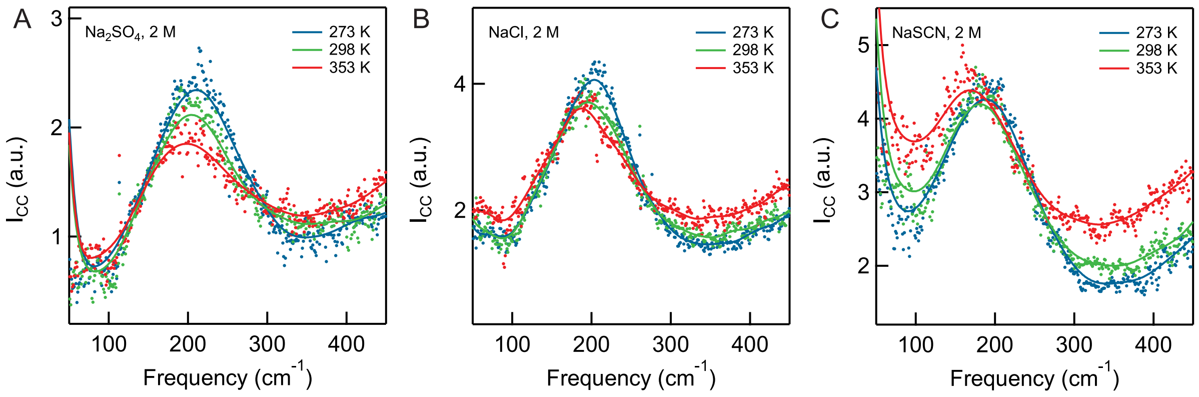

Figure S6 – Temperature dependence of the H-bond stretching mode in different 2 M salt solutions. I_{CC} spectra for 2 M Na_2SO_4 solutions (A), 2 M NaCl solutions (B) and 2 M NaSCN solutions (C), for three different temperatures 273 K (blue), 298 K (green) and 353 K (red). A strong temperature dependence is observed for Na_2SO_4 solutions – consistent with dominant electrostatic interactions, in contrast to smaller temperature dependence for NaSCN solutions – consistent with charge transfer induced covalency.

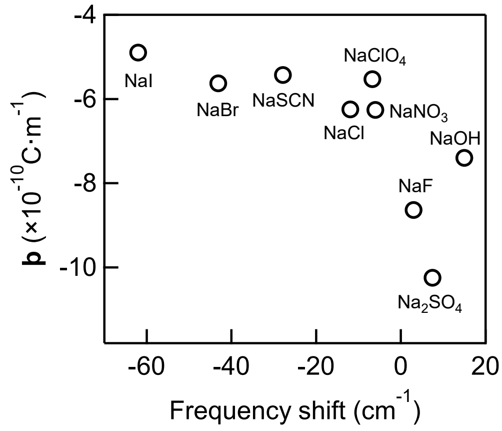

Figure S7 – Charge density as a function of the frequency shift in the H-bond stretching mode. The frequency shifts are from Fig.2 and the charge density is represented by the "sho" (**ϸ**) parameter defined in reference [21], and the **ϸ** values are obtained from there.

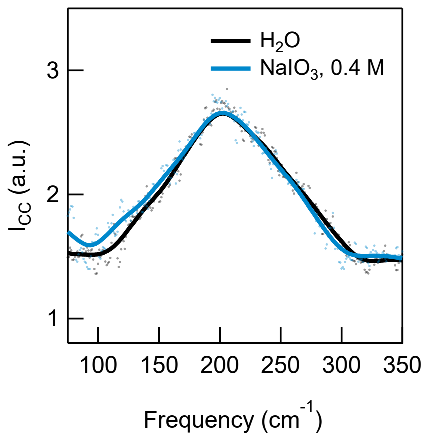

Figure S8 – CVS of sodium iodate solutions. I_{CC} spectrum of a 0.4 M $NaIO_3$ solution. An extremely small redshift is observed (~1 cm^{-1}) with no change in the intensity.

References

(1) Flor, M.; Wilkins, D. M.; de la Puente, M.; Laage, D.; Cassone, G.; Hassanali, A.; Roke, S. Dissecting the hydrogen bond network of water: Charge transfer and nuclear quantum effects. *Science* **2024**, *386* (6726), eads4369. DOI: 10.1126/science.ads4369.

(2) Yixing Chen, S. R. Generalized expressions for hyper-Rayleigh scattering from isotropic liquids. *arXiv:1705.04231 [physics.chem-ph]* **2017**, 12.

(3) Blazquez, S.; Conde, M. M.; Abascal, J. L. F.; Vega, C. The Madrid-2019 force field for electrolytes in water using TIP4P/2005 and scaled charges: Extension to the ions F-, Br-, I-, Rb+, and Cs+. *J Chem Phys* **2022**, *156* (4). DOI: Artn 04450510.1063/5.0077716.

(4) Abascal, J. L. F.; Vega, C. A general purpose model for the condensed phases of water: TIP4P/2005. *J Chem Phys* **2005**, *123* (23). DOI: Artn 23450510.1063/1.2121687.

(5) Abraham, M. J.; Murtola, T.; Schulz, R.; Páll, S.; Smith, J. C.; Hess, B.; Lindahl, E. GROMACS: High performance molecular simulations through multi-level parallelism from laptops to supercomputers. *SoftwareX* **2015**, *1-2*, 19-25. DOI: https://doi.org/10.1016/j.softx.2015.06.001.

(6) Darden, T.; York, D.; Pedersen, L. Particle Mesh Ewald - an N.Log(N) Method for Ewald Sums in Large Systems. *J Chem Phys* **1993**, *98* (12), 10089-10092. DOI: Doi 10.1063/1.464397.

(7) Bauer, P.; Hessand, B.; Lindahl, E. GROMACS 2022.5 Source code. Zenodo: https://doi.org/10.5281/zenodo.7586780, 2023.

(8) Berendsen, H. J. C.; Grigera, J. R.; Straatsma, T. P. The Missing Term in Effective Pair Potentials. *J Phys Chem-Us* **1987**, *91* (24), 6269-6271. DOI: DOI 10.1021/j100308a038.

(9) Essmann, U.; Perera, L.; Berkowitz, M. L.; Darden, T.; Lee, H.; Pedersen, L. G. A Smooth Particle Mesh Ewald Method. *J Chem Phys* **1995**, *103* (19), 8577-8593. DOI: Doi 10.1063/1.470117.

(10) Parrinello, M.; Rahman, A. Polymorphic Transitions in Single-Crystals - a New Molecular-Dynamics Method. *J Appl Phys* **1981**, *52* (12), 7182-7190. DOI: Doi 10.1063/1.328693.

(11) Hess, B.; Bekker, H.; Berendsen, H. J. C.; Fraaije, J. G. E. M. LINCS: A linear constraint solver for molecular simulations. *J Comput Chem* **1997**, *18* (12), 1463-1472. DOI: Doi 10.1002/(Sici)1096-987x(199709)18:12<1463::Aid-Jcc4>3.0.Co;2-H.

(12) Bussi, G.; Donadio, D.; Parrinello, M. Canonical sampling through velocity rescaling. *J Chem Phys* **2007**, *126* (1), 014101. DOI: 10.1063/1.2408420.

(13) Espinosa, E.; Molins, E.; Lecomte, C. Hydrogen bond strengths revealed by topological analyses of experimentally observed electron densities. *Chemical Physics Letters* **1998**, *285* (3-4), 170-173. DOI: Doi 10.1016/S0009-2614(98)00036-0.

(14) Schönfeldová, T.; Dupertuis, N.; Chen, Y.; Ansari, N.; Poli, E.; Wilkins, D. M.; Hassanali, A.; Roke, S. Charge Gradients around Dendritic Voids Cause Nanoscale Inhomogeneities in Liquid Water. *The Journal of Physical Chemistry Letters* **2022**, *13* (32), 7462-7468. DOI: 10.1021/acs.jpclett.2c01872.

(15) Schmidt, D. A.; Birer, Ö.; Funkner, S.; Born, B. P.; Gnanasekaran, R.; Schwaab, G. W.; Leitner, D. M.; Havenith, M. Rattling in the Cage: Ions as Probes of Sub-picosecond Water Network Dynamics. *Journal of the American Chemical Society* **2009**, *131* (51), 18512-18517. DOI: 10.1021/ja9083545.

(16) Smiechowski, M.; Sun, J.; Forbert, H.; Marx, D. Solvation shell resolved THz spectra of simple aqua ions - distinct distance- and frequency-dependent contributions of solvation shells. *Phys Chem Chem Phys* **2015**, *17* (13), 8323-8329. DOI: 10.1039/c4cp05268d.

(17) Kanamori, H.; Okamoto, H.; Urabe, K. Lattice vibrational modes in potassium thiocyanate. *Journal of Physics and Chemistry of Solids* **1981**, *42* (3), 197-202. DOI: https://doi.org/10.1016/0022-3697(81)90081-0.

(18) Funkner, S.; Niehues, G.; Schmidt, D. A.; Heyden, M.; Schwaab, G.; Callahan, K. M.; Tobias, D. J.; Havenith, M. Watching the Low-Frequency Motions in Aqueous Salt Solutions: The Terahertz Vibrational Signatures of Hydrated Ions. *Journal of the American Chemical Society* **2012**, *134* (2), 1030-1035. DOI: 10.1021/ja207929u.

(19) Schwaab, G.; Sebastiani, F.; Havenith, M. Ion Hydration and Ion Pairing as Probed by THz Spectroscopy. *Angewandte Chemie International Edition* **2019**, *58* (10), 3000-3013. DOI: 10.1002/anie.201805261.
(20) Israelachvili, J. N. *Intermolecular and surface forces*; Academic Press, 2010.
(21) Gregory, K. P.; Wanless, E. J.; Webber, G. B.; Craig, V. S. J.; Page, A. J. The electrostatic origins of specific ion effects: quantifying the Hofmeister series for anions. *Chem. Sci.* **2021**, *12* (45), 15007-15015. DOI: 10.1039/D1SC03568A.
